# Osseointegrative and antimicrobial properties of graphene oxide nano coated dental implants: A systematic review

**DOI:** 10.12688/f1000research.148180.2

**Published:** 2024-08-09

**Authors:** Sounyala Rayannavar, Sunil Kumar MV, Vignesh Kamath, Mahantesh Bembalgi, Namratha Nayak, Praveen Jodalli

**Affiliations:** 1 Department of Prosthodontics, KAHER's KLE Vishwanath Katti Institute of Dental Sciences, Belgavi, Karnataka, India; 2 Department of Prosthodontics, Jaipur Dental College, Maharaj Vinayak Global University, Jaipur, Rajasthan, India; 3 Department of Prosthodontics Crown and Bridge, Manipal college of Dental Sciences, Mangalore, Manipal Academy of Higher Education, Manipal, Karnataka, 576104, India; 4 Department of Prosthodontics, KAHER's KLE Vishwanath Katti Institute of Dental Sciences, Belgavi, Karnataka, India; 5 Department of Periodontology, Manipal College of Dental Sciences, Manipal Academy of Higher Education, Manipal, Karnataka, 576104, India; 6 Department of Public Health Dentistry, Manipal College of Dental Sciences, Mangalore, Manipal Academy of Higher Education, Manipal, Karnataka, 575001, India

**Keywords:** Graphene oxide, dental implants, osseointegration, antimicrobial properties, systematic review

## Abstract

**Introduction:**

Osseointegration stands as a pivotal concept within the realm of dental implants, signifying the intricate process through which a dental implant integrates with the adjoining bone tissue. Graphene oxide (GO) has been shown to promote osseointegration, the process by which the implant fuses with the surrounding bone. The objective of this study was to assess the osseointegrative and antimicrobial properties of GO nano coated dental implants.

**Methods:**

A systematic search was conducted using electronic databases (e.g., PubMed, Scopus, Web of Science) to identify relevant studies published. Inclusion criteria encompassed studies that evaluated the effects of GO nano coating on osseointegrative and antimicrobial characteristics of dental implants. Studies not written in English and published before 2012 were excluded.

**Results:**

The initial search yielded a total of 127 potential studies, of which six met the inclusion criteria and five were included in the review. These studies provided data on GO nano coated dental implants and their osseointegrative and antimicrobial properties. All the included studies showed moderate risk of bias. None of the studies provided information related to sample size calculation or sampling technique.

**Discussion:**

The findings from the included studies demonstrated that GO nano coating had a positive impact on osseointegrative properties of dental implants. Enhanced bone-implant contact and increased bone density were observed in animals and humans receiving GO nano coated implants. Furthermore, the antimicrobial properties of GO nano coating were found to inhibit bacterial colonization and biofilm formation on the implant surface, reducing the risk of implant-associated infections.

**Conclusion:**

The findings indicate that GO nano coating holds promise in enhancing the success rate and longevity of dental implants. However, more studies with larger sample sizes, are needed to further strengthen the evidence and determine the long-term effects of GO nano coated dental implants.

## Introduction

Osseointegration stands as a pivotal concept within the realm of dental implants, signifying the intricate process through which a dental implant integrates with the adjoining bone tissue. This integration results in the formation of a robust and enduring foundation for the replacement tooth or teeth. ^1^ This term is derived from the Latin words “Osseo” which means bone, and “integration,” which means to make whole. ^1^ Osseointegration primarily depends on the implant placement, healing period, biocompatibility, stability, longevity and maintenance of the dental implant. ^2^^,^^3^

The process of osseointegration begins when a dental implant, typically made of biocompatible materials like titanium, is surgically placed into the jawbone at the site of a missing tooth. This implant post serves as a replacement for the natural tooth’s root. Bio-activation as well as surface modification of titanium implants reduces an immune response and allows for successful osseointegration. ^4^ Cobalt chromium alloys, Iron-chromium-nickel based alloys and ceramics were also used as dental implant materials. Titanium alloys and titanium alloys stays superior to the previous dental implants due to its excellent osseointegrative properties. ^5^ While pure titanium implants and their alloys exhibit outstanding biocompatibility, mechanical strength, and chemical stability, they are associated with certain disadvantages, including delayed osseointegration and an extended period of post-operative healing. These factors can contribute to the potential failure of dental implants. ^6^ To address these limitations, implant surface alterations using techniques based on the immobilisation of biologically active organic compounds has been discovered. ^7^

Surface coating of titanium dental implants is an essential aspect of implant dentistry, as it plays a crucial role in enhancing osseointegration, stability, and long-term success of the implant. ^8^^,^^9^ There are several surface coatings and treatments that can be applied to titanium dental implants to improve their performance. Some of the commonly used surface coatings and treatments include: Anodic Oxidation (Anodization), Sandblasting and Acid Etching, Plasma Spraying, Blasted Surfaces, Titanium Nitride Coating, Zirconia Coating, Chemical Modifications. ^8^

Surface modifications using nanomaterials are widely used recently. These nanomaterials make good coating options for dental implants made of titanium (Ti-based) as it enhances implant fixation and encourages soft tissue integration and osteogenesis. In addition, osteoconductive nanoparticles creates a chemical bond with bone to ensure that implants are biologically fixed. ^10^ In order to achieve better osseointegrative properties, naturally bioactive and antimicrobial polymers like chitosan and graphene oxide have been employed. By exposing bioactive modifications to simulated bodily fluids, specific cellular and tissue reactions can be induced, leading to biomimetic precipitation of calcium phosphate (CaP). ^11^^–^^13^

Graphene coatings stimulate osteogenic differentiation, cell adhesion, and antibacterial activity. ^12^ Coating dental implants with graphene oxide can offer several potential benefits. Graphene oxide has demonstrated the ability to enhance osseointegration, the bonding process between the implant and the adjacent bone. The distinctive surface characteristics of graphene oxide promote enhanced bone adhesion, resulting in increased stability and prolonged success of the implant. Graphene oxide possesses antibacterial properties, which can help prevent infections around the implant site. ^14^ Graphene oxide is generally considered biocompatible. This means it is well-tolerated by the human body and does not provoke a significant immune response. The high surface area and unique properties of graphene oxide can be harnessed for controlled drug delivery. This may allow for the local release of antibiotics or other therapeutic agents to further prevent infection and promote healing. Graphene oxide coatings can improve the visibility of dental implants in imaging techniques like X-rays or CT scans, aiding in diagnosis and follow-up care. ^15^^–^^17^ Their extensive surface area and ability to be functionalized allow for precise drug delivery, enhancing drug effectiveness and minimizing side effects. The amalgamation of graphene oxide with other biomaterials can augment the surface properties of a material, impart antibacterial attributes, and influence cellular behavior. ^15^^,^^16^

Various studies have been conducted to compare the osteogenic potential, physical properties and biologic properties of titanium dental implants with or without graphene oxide surface modification. ^18^^–^^20^ As these comparative studies conducted are on different forms of surface coatings on titanium implants, the current systematic review aimed to examine and analyse osseointegrative properties of graphene oxide coated Titanium dental implants compared to conventional titanium dental implants without any surface modifications.

## Methods

The current review was conducted in accordance with PRISMA (Preferred Reporting Items for Systematic Reviews and Meta-Analyses) guidelines. ^21^ This review has been officially registered with PROSPERO and has been assigned the registration number CRD42023413883.

### Search strategy

We performed an extensive search across multiple electronic databases, including PubMed, Scopus, Web of Science, and Embase, to identify relevant articles evaluating the osteointegration and biological properties of titanium dental implants coated with graphene oxide. The search utilized key terms such as “graphene oxide,” “titanium implants,” “osseointegration,” “surface roughness,” and “surface characteristics,” combined using Boolean operators “AND” or “OR” to optimize article retrieval. The inclusion criteria for this review encompassed studies published from January 1, 2012, to December 31, 2022, covering a span of 10 years. The rationale behind this timeframe was to focus on the recently emerged method of surface modification involving graphene oxide coating on titanium dental implants. Consequently, studies published before 2012 were excluded. ^20^ The details of search strategy are mentioned in Table 1. ^22^ Details of the studies were uploaded in figshare open repository and publically available. ^22^

**Table 1.** Search strategy.

(“titanium”[MeSH Terms] OR “titanium”[All Fields] OR “titaniums”[All Fields]) AND (“dental implants”[MeSH Terms] OR (“dental”[All Fields] AND "implants”[All Fields]) OR “dental implants”[All Fields]) AND ((“surface”[All Fields] OR “surface s”[All Fields] OR “surfaced”[All Fields] OR “surfaces”[All Fields] OR “surfacing”[All Fields] OR “surfacings”[All Fields]) AND (“coated”[All Fields] OR “coating”[All Fields] OR “coating s”[All Fields] OR “coatings”[All Fields])) AND (“graphene oxide”[Supplementary Concept] OR “graphene oxide”[All Fields]) AND (“osseointegrate”[All Fields] OR “osseointegrated”[All Fields] OR “osseointegrates”[All Fields] OR “osseointegrating”[All Fields] OR “osseointegration”[MeSH Terms] OR “osseointegration”[All Fields] OR “osseointegrative”[All Fields])

### Study selection

The studies included were in line with the PICO (Population, Intervention, Comparison and Outcomes) criteria ^23^:

• Population: Titanium dental implants
• Intervention(s): surface modification of titanium dental implants with graphene oxide coating
• Comparator(s)/control: Conventional implant or surface modification done with other materials such as chitosan
• Outcome(s): Osseointegration and biological properties of Graphene oxide nano coated titanium dental implants.

### Inclusion criteria

**Figure 1.**
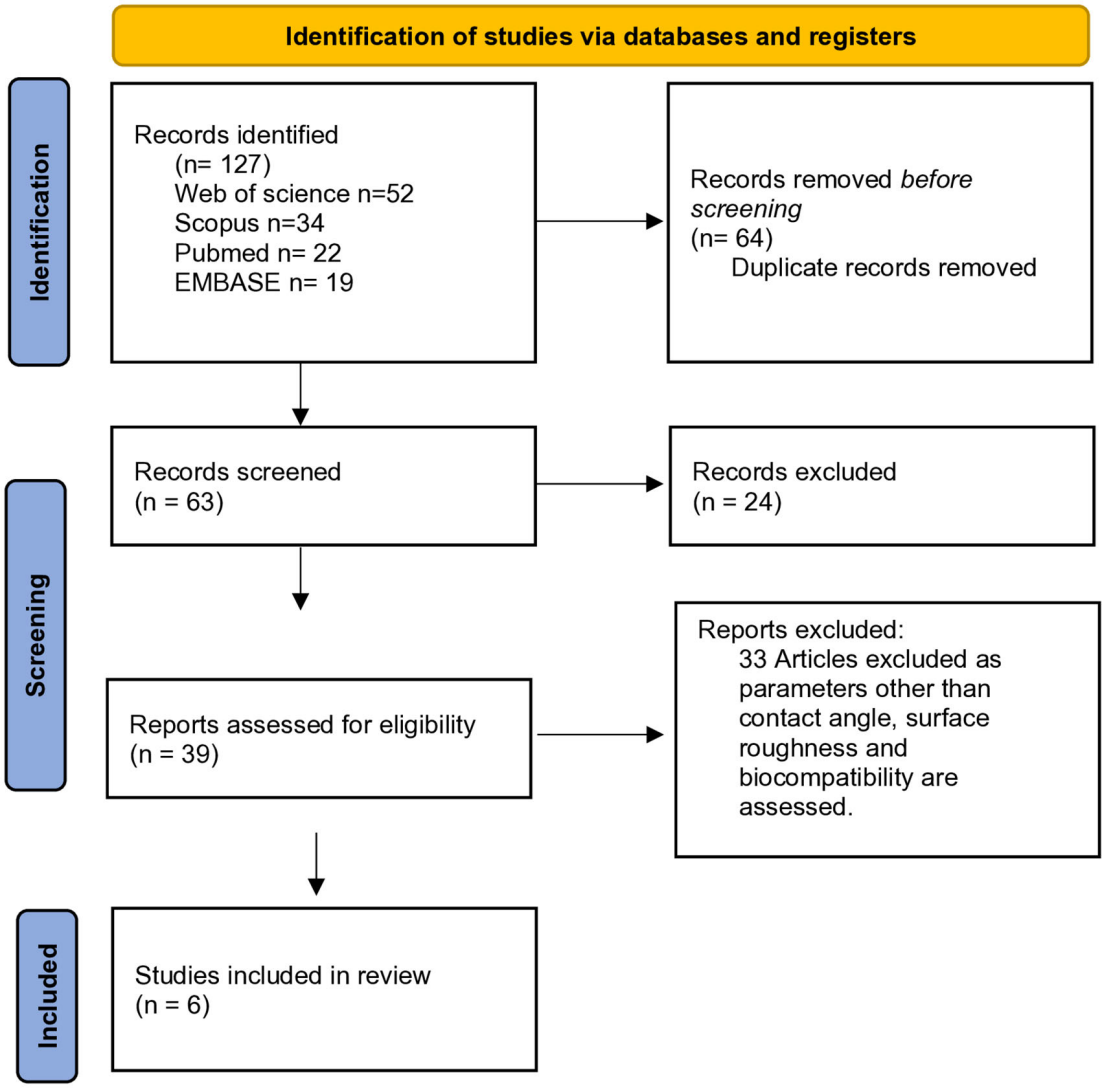
PRISMA flowchart describing the inclusion process of articles.

Two reviewers (SR and VK) independently evaluated titles and abstracts to identify potentially suitable studies. Any uncertainties were resolved through discussion with a third reviewer (SK). The inclusion criteria for the chosen studies encompassed English language research articles with complete full-text manuscripts. Additionally, all in vitro studies, clinical trials, randomized clinical trials, comparative studies, and cross-sectional studies relevant to the topic were considered for inclusion, while case reports, case series, narrative reviews, and single-arm longitudinal studies were excluded. Abstracts without accompanying full-text articles were also excluded from the conduct and reporting of the present systematic review . Flowchart illustrating the process for inclusion of articles is illustrated in Figure 1.

### Data extraction

The articles obtained following the literature search underwent scrutiny by the study investigators to eliminate duplicated or irrelevant studies. Subsequently, two reviewers (SR and VK) independently conducted further screening of the remaining articles to assess their eligibility for inclusion in the systematic review. For each study, the following factors were meticulously noted: Author name(s), study design, type of coating, method of coating, surface characteristics, mechanical properties, cell adhesion, and antimicrobial properties. ^22^

### Quality assessment

Risk of Bias assessment: The quality assessment of the studies included in the review was conducted using the QUIN tool specifically designed for evaluating the risk of bias in in-vitro studies. ^24^

This tool assesses bias across 12 domains, encompassing clearly stated aims and objectives, a comprehensive explanation of sample size calculation, detailing of the sampling technique, information about the comparison group, methodology explanation, operator details, randomization process, method of outcome measurement, details of the outcome assessor, blinding, statistical analysis, and presentation of results. ^22^^,^^24^

## Results

The current systematic review focused on determining whether osseointegration and the biological properties of graphene oxide nano coated titanium dental implants are superior to conventional or other nanomaterial-coated titanium dental implants. The study entailed a comparative analysis of contact angle and surface roughness between conventional/chitosan-coated titanium implants and graphene oxide-coated titanium implants.

### Study selection

The electronic search was conducted through three distinct stages. In the initial stage, a total of 127 articles were initially retrieved by employing specified keywords in the electronic databases. Two independent reviewers (VK and SR) conducted this search. The titles of the retrieved studies were then screened to ensure they met the eligibility criteria outlined by the PICO framework. Moving on to the second stage, abstracts of all selected titles were meticulously screened, and the complete texts of 39 studies deemed potentially pertinent were identified for the third stage. Notably, 33 studies were excluded at this stage as they assessed parameters beyond contact angle, surface roughness, and biocompatibility.

In the third stage, a detailed review was conducted on the complete texts of the 39 identified studies. Ultimately, 6 studies were selected for inclusion in this review. ^12^^,^^25^^–^^29^ One of the reviewed articles exhibited a discrepancy in measurement presentation for outcomes (contact angle and surface roughness), resulting in the exclusion of these studies from the quantitative analysis ^12^ ( Table 2).

**Table 2.** characteristics of the studies included.

Sl. No.	Study ID	Implant type	Surface characteristics			Cell proliferation	ALP activity	Biofilm thickness	Other outcomes
			Spectra	Contact angle	Surface roughness				
1	Corado et al 2022 ^12^	1.Ti 2. Ti-GO 3. Ti-NB. 4. Ti-NBGO.	EDS spectra	1.Ti: 50.74 ± 4.6 2. Ti-GO: 100.35 ±10.85 3. Ti-NB.: 44.33 ± 10.0 4. Ti-NBGO: 55.86 ± 6.1	0.52 ± 0.06 0.44 ± 0.03 0.47 ± 0.04 0.64 ± 0.47	NA	NA	NA	Surface energy 51.61 ± 4.79 40.80 ± 13.47 12.94 ± 9.33 46.10 ± 6.38
2	Shin et al 2022 ^25^	1. ST (control) 2. rhBMP-2-immobilized ST (BI-ST), 3. rhBMP-2-treated ST (BT-ST), 4. rGO-coated ST (R-ST).	Raman shift	ST: 79.3 ± 0.9 nm R-ST discs: 7.1 ± 1.8 nm		21 days: ST: 400 BI-ST: 395 BT-ST: 395 R-ST: 500	21 days: ST: 15 BI-ST: 25 BT-ST: 50 R-ST: 98		Cell attachment: ST: 100 BI-ST: 100 BT-ST: 120 R-ST: 150
3	Guo et al 2021 ^26^	1. PEEK (P) 2. PEEK PDA (PA) 3. PEEK PDA GO (PAG)	PAG coated PEEK materials	1. P: 83.5 ^°^ ± 0.74 2. PA: 68 ^°^ ±3.1 3. PAG: 53 ^°^ ±					
4	Kang et al 2021 ^27^	1. Intact Ti 2. rGO Ti	rGO Ti showed strong Raman peak successfully coated rGO on Ti substrates	1. Intact Ti: 127.4 ± 1.0 2. rGO Ti: 76.3 ± 2.4	Surface energy 1. Intact Ti: 8.0 ± 0.5 2. rGO Ti: 37.7 ± 1.1	1. Intact Ti: 400 2. rGO Ti: 500	1. Intact Ti: 10 2. rGO Ti: 25		Mineralization: 1. Intact Ti: 250 2. rGO Ti: 1000
5	Park et al 2020 ^28^	1. Chitosan implant 2. Graphene chitosan 1% 3% 5%	Surfaces of hybrid Implants showed GO integration compared to Chitosn implants.	1. Chitosan: 61.5° 2. GC 1%: 56.2°, 3% GC: 54.6°, and 5% GC: 54.5°.	1. Chitosan: 114.6 ± 7.9 nm 2. GC 1%: 124.6 ± 4.7 nm 3% GC: 140.7 ± 8.8 nm and 5% GC: 149.0 ± 7.2 nm	1. Chitosan implant: 1 abr unit 2. GC: 1%: 1.8 3%: 1.5 5%: 1.5		1. Chitosan: 18 m 2. Graphene chitosan 1%: 10m 3%: 15m 5%: 10m05	Antibacterial effect: 1% GC > 3% GC > 5% GC > chitosan)
6	Mazaheri et al 2014 ^29^	1. Chitosan 2. GO(1.5 wt%)–chitosan 3. GO(3 wt%)–chitosan 4. GO(6 wt%)–chitosan			1. Chitosan: 1.6 ± 0.1 2. GO(1.5 wt%) chitosan: 2.4 ± 0.2 3. GO(3 wt%)–chitosan: 12.9 ± 1.1 4. GO(6 wt%+)–chitosan: 17.5 ± 2.9			NA	

### Risk of bias assessment

Every study included in the analysis demonstrated a moderate risk of bias. None of the studies furnished details pertaining to sample size calculation or the employed sampling technique. Details of blinding of the examiners was unclear in the included studies ( Table 3).

**Table 3.** Risk of bias.

Study ID	Clearly stated aim, objectives	Detailed explanation of sample size calculation	Detailed explanation of sampling technique	Details of comparison group	Detailed explanation of methodology	Operator details	Randomization	Method of outcome measurement	Outcome assessment details	Blinding	Statistical analysis	Presentation of results	Risk of bias
Corado et al 2022 ^12^	2	1	0	2	2	2	0	0	2	0	2	2	Moderate
Shin et al 2022 ^25^	2	0	0	1	2	2	0	1	2	0	2	2	Moderate
Guo et al 2021 ^26^	2	0	0	2	2	2	0	0	2	0	2	2	Moderate
Kang et al 2021 ^27^	2	0	0	2	2	2	0	0	2	0	2	2	Moderate
Park et al 2020 ^28^	2	0	0	1	2	2	0	2	2	0	2	2	Moderate
Mazaheri et al 2014 ^29^	2	0	0	2	2	2	0	0	2	0	2	2	Moderate

## Discussion

Graphene oxide has recently demonstrated remarkable achievements in dentistry, spanning the treatment of oral cancer, regenerative dentistry, drug delivery, and antibacterial applications. ^27^ Liu et al. (2011) conducted a review in the domain of oral disease treatment, emphasizing the potential application of graphene oxide as a restorative material for addressing dental caries. The review underscored the utilization of graphene oxide due to its superior physicochemical and mechanical characteristics. Furthermore, the study highlighted its compatibility with glass ionomer cements, demonstrating the capability to improve the mechanical properties of composites without compromising their aesthetic attributes or their ability to release fluoride. ^30^

Graphene and graphene oxide have demonstrated characteristics with potential anticaries effects, inhibiting the colonization of *S. mutans* and *P. gingivalis*. Moreover, these materials exhibit the capability to stimulate the differentiation and proliferation of human dental pulp stem cells (hDPSC) and stem cells from periodontal ligaments (PDLSC). This property is conducive to the regeneration of dental pulp and periodontal ligament tissues. ^29^^,^^31^

Graphene oxide finds extensive use in dental implants owing to its notable surface energy, mechanical strength, and biointegration properties. The incorporation of graphene in implants is primarily driven by its capacity to physically interact with biomolecules such as enzymes, proteins, or peptides. Graphene exhibits exceptional biocompatibility, efficiently stimulates and matures stem cells, and possesses long-term durability. Furthermore, the substantial surface area of graphene is a crucial aspect with significant potential for future bio-functionalization. ^30^ Titanium implants are coated with both graphene oxide and reduced graphene oxide (rGO) showcased their inherent ability to induce osteogenic differentiation. This characteristic is of particular significance for the long-term success of dental implants in comparison to conventional surface treatments like grit-blasting, acid etching, and micro-arc oxidation. ^32^^,^^33^

The analysis focused on studies that evaluated Osseointegration properties, utilizing available data from the selected studies, including contact angle and surface roughness. Additionally, the application of reduced graphene oxide as a surface coating in titanium implants was also explored. Both reduced graphene oxide and graphene oxide coating renders similar surface characteristics to the titanium implant. Other characteristics such as Raman Shift, surface energy and biologic properties (cell proliferation, Alkaline Phosphatase [ALP] activity and biofilm thickness were not assessed as the study results of these parameters were presented in different measurements (either in percentages or in abr units) and at different time duration. Hence the quantitative analysis of same was not performed due to the differences in data presentation. However, study by Shin et al.,2022 showed that reduced graphene oxide nanocoated titanium dental implants have high cell proliferation rate and ALP activity within 21 days. ^25^ Cell attachment was also observed to be higher in reduced graphene oxide coated titanium implants. Kang et al., 2021 also reported a higher mineralization with reduced graphene oxide coated titanium dental implants. ^27^

**Figure 2.**
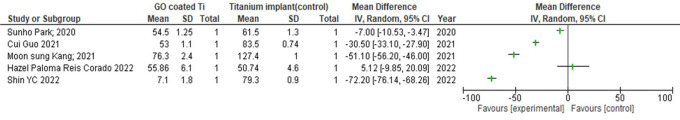
Comparison of contact angle for GO coated Ti implants and Ti implants.

**Figure 3.**
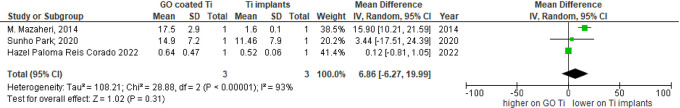
Comparison of surface roughness for GO coated Ti implants and Ti implants.

Quantitative assessment of the selected studies was conducted to evaluate surface characteristics, specifically surface roughness and contact angle are represented in Figure 2 and Figure 3. ^22^ In the comparison between Graphene Oxide-coated titanium implants and Chitosan/control implants, the latter exhibited a lower contact angle and higher surface roughness. These findings suggest enhanced adhesion to the periosteum. Notably, research has demonstrated that reduced graphene oxide coating, when combined with concentrated growth factors, facilitates the differentiation of implants into osteoblasts. This acknowledgment highlights its potential as a revolutionary material for altering dental implants and acting as a foundational structure for the regeneration of bone tissue.

Shin et al. and Kwak et al. suggested that increased rGO concentration on the surfaces of titanium implants leads to rougher surfaces capable of absorbing exogenous proteins, thereby promoting cell proliferation and osteogenic differentiation. ^25^^,^^34^

The recognized antibacterial mechanisms of GO currently include the physical breakdown of the cell membrane and the induction of damage through oxidative stress. Generally, materials based on graphene are known to generate oxidative stress, primarily through reactive oxygen species, resulting in antibacterial effects and significant harm to bacterial cells. ^19^ Another antibacterial mechanism of GO involves the dispersibility and trapping capacity of its oxygen-containing functional groups. ^35^ In accordance with the insights from the review, the reduction of graphene oxide has been observed to enhance antibacterial capabilities by diminishing bacterial colonization on graphene oxide-coated titanium implants. Notably, a substantial enhancement in osseointegrative characteristics has been evident, as evidenced by elevated alkaline phosphatase activity and increased cell proliferation around the graphene oxide-coated titanium implants. These findings underscore the multifaceted benefits of optimizing graphene oxide configurations for improved antimicrobial and osseointegrative performance in the context of dental implants. The hydrophobic nature of graphene oxide plays a role in preventing bacterial cells from adhering to each other, and this hydrophobic contact can lead to the breakdown of the bacterial membrane, exerting an antibiotic effect. ^13^^,^^36^^,^^37^

The expression of ALP, and extracellular matrix proteins such as RUNX2 and COL1A1 that promotes osteoblast differentiation appears to be improved by rGO as well. ^38^ Several studies have shown that through the activation of the focal adhesion kinase (FAK)/P38 pathway, improved bone mesenchymal stem cells (BMSC) adhesion capacity, and proliferation, GO can trigger early osteogenic differentiation of BMSCs. ^39^ Additionally, hMSC (human mesenchymal stem cells) adhesion, migration, and proliferation can be controlled by graphene oxide. ^40^^–^^42^ Hence graphene oxide coated dental implants serves higher chances in success of dental implants due to its improved biological and mechanical properties compared to conventional/chitosan coated dental implants. ^43^ Moreover, it is imperative to conduct clinical trials and studies to thoroughly evaluate the biocompatibility, mechanical properties, and long-term performance of graphene oxide-coated titanium dental implants, ensuring a comprehensive understanding of their advanced properties.

Considering the scarcity in in-vitro studies conducted in comparing the biological properties and antimicrobial properties of graphene oxide surface coated titanium dental implants, more studies could be conducted in future. Studies comparing graphene oxide coated titanium dental implants with other bioactive surface modification materials such as ceramic and composites would also determine the superiority of biological, mechanical and antimicrobial properties of graphene oxide surface coating.

## Conclusion

Utilizing graphene oxide as a coating for titanium implants creates a highly biocompatible surface, promoting enhanced integration with surrounding tissues and reducing the risk of rejection or complications. The exceptional mechanical strength and flexibility inherent in graphene oxide further contribute to the durability and longevity of the implants, ensuring not only effective integration but also sustained long-term performance. The success or failure of dental implants hinges on various factors, including implant location, the load-bearing capacity of the underlying bone, and the overall health of the patient.

In conclusion, the potential transformation of implantology is embodied in graphene oxide-coated titanium dental implants, driven by advancements in biocompatibility, osseointegration, antibacterial properties, and controlled drug delivery. Continued research and thorough scientific inquiry have the potential to position these implants as significant contributors to considerably improved patient outcomes, a reduction in complications, and an overall enhancement in the quality of life for individuals in need of dental implants.

## Authors’ contributions

All authors contributed equally for this study and preparation of manuscript.

## Data Availability

All underlying data are available as part of the article and no additional source data are required. Figshare: Osseointegrative and antimicrobial properties of graphene oxide nano coated dental implants: A systematic review, https://doi.org/10.6084/m9.figshare.25163666.v1. ^22^ Data are available under the terms of the Creative Commons Attribution 4.0 International license (CC-BY 4.0). Figshare: checklist for Osseointegrative and antimicrobial properties of graphene oxide nano coated dental implants: A systematic review, https://doi.org/10.6084/m9.figshare.25163666.v1. ^22^

## References

[1] Jayesh RS Dhinakarsamy V : Osseointegration. *J. Pharm. Bioallied Sci.* 2015 Apr;7(Suppl 1):226–229. 10.4103/0975-7406.155917 26015719 PMC4439679

[2] Parithimarkalaignan S Padmanabhan TV : Osseointegration: an update. *J. Indian Prosthodont. Soc.* 2013 Mar;13(1):2–6. Epub 2013 Jan 11. 10.1007/s13191-013-0252-z 24431699 PMC3602536

[3] Le Guéhennec L Soueidan A Layrolle P : Surface treatments of titanium dental implants for rapid osseointegration. *Dent. Mater.* 2007 Jul;23(7):844–854. 10.1016/j.dental.2006.06.025 16904738

[4] Silva RCS Agrelli A Andrade AN : Titanium Dental Implants: An Overview of Applied Nanobiotechnology to Improve Biocompatibility and Prevent Infections. *Materials.* 2022 Apr 27;15(9):3150. 10.3390/ma15093150 35591484 PMC9104688

[5] Saini M Singh Y Arora P : Implant biomaterials: A comprehensive review. *World J. Clin. Cases.* 2015 Jan 16;3(1):52–57. 10.12998/wjcc.v3.i1.52 25610850 PMC4295219

[6] Tribst JPM Werner A Blom EJ : Failed Dental Implant: When Titanium Fractures. *Diagnostics.* 2023;13(12):2123. 10.3390/diagnostics13122123 37371017 PMC10297549

[7] Jemat A Ghazali MJ Razali M : Surface Modifications and Their Effects on Titanium Dental Implants. *Biomed. Res. Int.* 2015;2015:791725. Epub 2015 Sep 7. 10.1155/2015/791725 26436097 PMC4575991

[8] Vishnu S Kusum D : Advances in surface modification of dental implants from micron to nanotopography. *Int. J. Res. Dent.* 2011;1:1–10.

[9] Pandey C Rokaya D Bhattarai BP : Contemporary Concepts in Osseointegration of Dental Implants: A Review. *Biomed. Res. Int.* 2022;2022:6170452. 35747499 10.1155/2022/6170452 PMC9213185

[10] Parnia F Yazdani J Javaherzadeh V : Overview of Nanoparticle Coating of Dental Implants for Enhanced Osseointegration and Antimicrobial Purposes. *J. Pharm. Pharm. Sci.* 2017 May 29;20:148–160. 10.18433/J3GP6G 28554344

[11] Zhang Y Gulati K Li Z : Dental Implant Nano-Engineering: Advances, Limitations and Future Directions. *Nanomaterials (Basel).* 2021 Sep 24;11(10):2489. 10.3390/nano11102489 34684930 PMC8538755

[12] Corado HPR Souza M de Soraes F : Titanium Coated with Graphene and Niobium Pentoxide for Biomaterial Applications. *Int. J. Biomater.* 2022 Nov 30;2022:2786101–2786111. 10.1155/2022/2786101 36506263 PMC9729051

[13] Tan J Li L Li B : Titanium Surfaces Modified with Graphene Oxide/Gelatin Composite Coatings for Enhanced Antibacterial Properties and Biological Activities. *ACS Omega.* 2022 Jul 25;7(31):27359–27368. 10.1021/acsomega.2c02387 35967064 PMC9366957

[14] La WG Jin M Park S : Delivery of bone morphogenetic protein-2 and substance P using graphene oxide for bone regeneration. *Int. J. Nanomedicine.* 2014 May 7;107–107. 10.2147/IJN.S50742 24872706 PMC4024979

[15] Homas A Johnson JA : Graphene-Based Materials in Dental Applications: Antibacterial, Biocompatible, and Bone Regenerative Properties. Seifalian A, editor. *Int. J. Biomater.* 2023 Feb 7;2023:1–18. 10.1155/2023/8803283 PMC9929215 36819211

[16] Al-Noaman A Rawlinson SCF : A novel bioactive glass/graphene oxide composite coating for a polyether ether ketone-based dental implant. *Eur. J. Oral Sci.* 2023 Apr;131(2):e12915. Epub 2023 Jan 27. 10.1111/eos.12915 36707252

[17] Srimaneepong V Skallevold HE Khurshid Z : Graphene for Antimicrobial and Coating Application. *Int. J. Mol. Sci.* 2022;23(1). 10.3390/ijms23010499 35008923 PMC8745297

[18] Jang W Kim HS Alam K : Direct-Deposited Graphene Oxide on Dental Implants for Antimicrobial Activities and Osteogenesis. *Int. J. Nanomedicine.* 2021;16:5745–5754. 10.2147/IJN.S319569 34471350 PMC8404087

[19] Özcan M Volpato CAM Hian L : Graphene for Zirconia and Titanium Composites in Dental Implants: Significance and Predictions. *Curr. Oral Health Rep.* 2022;9:66–74. 10.1007/s40496-022-00310-3

[20] Mobarak MH Hossain N Hossain A : Advances of graphene nanoparticles in dental implant applications – A review. *Appl. Surf. Sci. Adv.* 2023 Dec 1 [cited 2023 Nov 25];18:100470. 10.1016/j.apsadv.2023.100470 Reference Source

[21] Page MJ McKenzie JE Bossuyt PM : The PRISMA 2020 statement: an updated guideline for reporting systematic reviews. *BMJ.* 2021;372:n7. 10.1136/bmj.n71 33782057 PMC8005924

[22] Rayannavar S Kumar MVS Kamath V : Osseointegrative and antimicrobial properties of graphene oxide nano coated dental implants: A systematic review. *figshare.* 2024 [cited 2024Mar29]. Reference Source 10.12688/f1000research.148180.2 PMC11325139 39149510

[23] Aslam S Emmanuel P : Formulating a Researchable question: a Critical Step for Facilitating Good Clinical Research. *Indian J. Sex Transm. Dis. AIDS.* 2010;31(1):47–50. 10.4103/0253-7184.69003 21808439 PMC3140151

[24] Sheth VH Shah NP Jain R : Development and validation of a risk-of-bias tool for assessing in vitro studies conducted in dentistry: The QUIN. *J. Prosthet. Dent.* 2022 Jun. 10.1016/j.prosdent.2022.05.019 35752496

[25] Shin YC Bae JH Lee JH : Enhanced osseointegration of dental implants with reduced graphene oxide coating. *Biomater. Res.* 2022 Mar 21;26(1):11. 10.1186/s40824-022-00257-7 35313996 PMC8935794

[26] Guo C Lu R Wang X : Graphene Oxide-Modified Polyetheretherketone with Excellent Antibacterial Properties and Biocompatibility for Implant Abutment. *Macromol. Res.* 2021 May;29(5):351–359. 10.1007/s13233-021-9042-3

[27] Kang MS Jeong S Lee SH : Reduced graphene oxide coating enhances osteogenic differentiation of human mesenchymal stem cells on Ti surfaces. 2021 Feb 12;25(1). 10.1186/s40824-021-00205-x PMC7881470 33579390

[28] Park S Kim HR Choi KS : Graphene–Chitosan Hybrid Dental Implants with Enhanced Antibacterial and Cell-Proliferation Properties. 2020 Jul 16;10(14):4888–4888.

[29] Mazaheri M Akhavan O Simchi A : Flexible bactericidal graphene oxide–chitosan layers for stem cell proliferation. *Appl. Surf. Sci.* 2014 May;301:456–462. 10.1016/j.apsusc.2014.02.099

[30] Liu S Zeng TH Hofmann M : Antibacterial Activity of Graphite, Graphite Oxide, Graphene Oxide, and Reduced Graphene Oxide: Membrane and Oxidative Stress. *ACS Nano.* 2011 Aug 24;5(9):6971–6980. 10.1021/nn202451x 21851105

[31] Nizami MZI Takashiba S Nishina Y : Graphene oxide: A new direction in dentistry. *Appl. Mater. Today.* 2020 Jun;19:100576. 10.1016/j.apmt.2020.100576

[32] Lee JH Shin YC Jin OS : Reduced graphene oxide-coated hydroxyapatite composites stimulate spontaneous osteogenic differentiation of human mesenchymal stem cells. *Nanoscale.* 2015;7(27):11642–11651. 10.1039/C5NR01580D 26098486

[33] Li X Lin K Wang Z : Enhanced growth and osteogenic differentiation of MC3T3-E1 cells on Ti6Al4V alloys modified with reduced graphene oxide. *RSC Adv.* 2017;7:14430–14437. 10.1039/C6RA25832H

[34] Kwak JM Kim J Lee C-S : Graphene Oxide as a Biocompatible and Osteoinductive Agent to Promote Implant Osseointegration in a Rabbit Tibia Model. *Adv. Mater. Interfaces.* 2022;9:2201116. 10.1002/admi.202201116

[35] Ghensi P Bressan E Gardin C : Osteo Growth Induction titanium surface treatment reduces ROS production of mesenchymal stem cells increasing their osteogenic commitment. *Mater. Sci. Eng. C.* 2017 May 1;74:389–398. 10.1016/j.msec.2016.12.032 28254309

[36] Li X Liang X Wang Y : Graphene-Based Nanomaterials for Dental Applications: Principles, Current Advances, and Future Outlook. 2022 Mar 10 [cited 2023 Jul 3];10. Reference Source 10.3389/fbioe.2022.804201 PMC8961302 35360406

[37] Zou X Zhang L Wang Z : Mechanisms of the antimicrobial activities of graphene materials. *J. Am. Chem. Soc.* 2016;138(7):2064–2077. 10.1021/jacs.5b11411 26824139

[38] Komori T ; Regulation of bone development and extracellular matrix protein genes by RUNX2. *Cell Tissue Res.* 2010 Jan;339(1):189–95. Epub 2009 Aug 1. 10.1007/s00441-009-0832-8 19649655

[39] Li Q Wang Z : Involvement of FAK/P38 Signaling Pathways in Mediating the Enhanced Osteogenesis Induced by Nano-Graphene Oxide Modification on Titanium Implant Surface. *Int. J. Nanomedicine.* 2020 Jun 30;15:4659–4676. 10.2147/IJN.S245608 32636624 PMC7335313

[40] Akhavan O Ghaderi E : Escherichia coli bacteria reduce graphene oxide to bactericidal graphene in a self-limiting manner. *Carbon.* 2012;50(5):1853–1860. 10.1016/j.carbon.2011.12.035

[41] Lu J Sun J Zou D : Graphene-Modified Titanium Surface Enhances Local Growth Factor Adsorption and Promotes Osteogenic Differentiation of Bone Marrow Stromal Cells. *Front. Bioeng. Biotechnol.* 2021 Jan 12;8:621788. 10.3389/fbioe.2020.621788 33511107 PMC7835422

[42] Docheva D Popov C Mutschler W : Human mesenchymal stem cells in contact with their environment: surface characteristics and the integrin system. *J. Cell. Mol. Med.* 2007 Jan-Feb;11(1):21–38. 10.1111/j.1582-4934.2007.00001.x 17367499 PMC4401218

[43] Daulbayev C Sultanov F Korobeinyk AV : Effect of graphene oxide/hydroxyapatite nanocomposite on osteogenic differentiation and antimicrobial activity. *Surf. Interfac.* 2022 Feb 1;28:101683–101683. 10.1016/j.surfin.2021.101683

