# Supplementary material for: Osseointegrative and antimicrobial properties of graphene oxide nano coated dental implants: A systematic review
Source: F1000Res. 2024 Aug 9;13:281. Originally published 2024 Apr 17. [Version 2] doi: 10.12688/f1000research.148180.2 (PMC11325139; doi:10.12688/f1000research.148180.2)
Supplement: Peer review history

# Reviewer report: Reviewer response for version 2

**DOI:** 10.5256/f1000research.170004.r313031

**Published:** 2024-09-13

**Authors:** Pooja Latti

**Affiliations:** 1 Department of Public Health Dentistry, Anoor Dental college and Hospital, Muvattupuzha, Kerala, India

**Abstract:**

*Methodology: Mention the exact time frame of the studies. To add on the search terms and data collected from the studies.*

*The results section to be modified with the relevant observations made from the reviewed studies. The number of studies included has to be made clear.*

**Introduction:**

 Graphene coatings stimulate osteogenic differentiation, cell adhesion, and antibacterial activity. ^12^ - T *o cite additional supporting references.*

 Various studies have been conducted to compare the osteogenic potential, physical properties and biologic properties of titanium dental implants with or without graphene oxide surface modification. ^18^^–^^20 -^ *Reference number 19 and 20 are review articles and not a research article. Hence requested to cite  relevent research articles.*

***Discussion:***

 Several studies have shown that through the activation of the focal adhesion kinase (FAK)/P38 pathway, improved bone mesenchymal stem cells (BMSC) adhesion capacity, and proliferation, GO can trigger early osteogenic differentiation of BMSCs. ^39 ^- *To cite the supporting studies.*

Are the rationale for, and objectives of, the Systematic Review clearly stated?

Yes

Is the statistical analysis and its interpretation appropriate?

I cannot comment. A qualified statistician is required.

If this is a Living Systematic Review, is the ‘living’ method appropriate and is the search schedule clearly defined and justified? (‘Living Systematic Review’ or a variation of this term should be included in the title.)

Not applicable

Are sufficient details of the methods and analysis provided to allow replication by others?

Yes

Are the conclusions drawn adequately supported by the results presented in the review?

Yes

Reviewer Expertise:

Public Health, Oral Health, Ethics, Preventive Dentistry

I confirm that I have read this submission and believe that I have an appropriate level of expertise to confirm that it is of an acceptable scientific standard, however I have significant reservations, as outlined above.

# Reviewer report: Reviewer response for version 2

**DOI:** 10.5256/f1000research.170004.r313026

**Published:** 2024-08-14

**Authors:** Vikram Garcha

**Affiliations:** 1 Sinhgad Dental College and Hospital, Pune, Maharashtra, India

1. The authors have made a good attempt in writing the manuscript regarding to study and evaluate the oseo-integrative and the  antimicrobial properties of graphene on dental implants.

 2. The authors can consider adding the details of favours control and favours experimental in Fig 2 as done for fig 3.

 3. Best wishes

Are the rationale for, and objectives of, the Systematic Review clearly stated?

Yes

Is the statistical analysis and its interpretation appropriate?

Yes

If this is a Living Systematic Review, is the ‘living’ method appropriate and is the search schedule clearly defined and justified? (‘Living Systematic Review’ or a variation of this term should be included in the title.)

Yes

Are sufficient details of the methods and analysis provided to allow replication by others?

Yes

Are the conclusions drawn adequately supported by the results presented in the review?

Yes

Reviewer Expertise:

Research and Statistics, Health informatics, Health education.

I confirm that I have read this submission and believe that I have an appropriate level of expertise to confirm that it is of an acceptable scientific standard.

# Reviewer report: Reviewer response for version 2

**DOI:** 10.5256/f1000research.170004.r312736

**Published:** 2024-08-12

**Authors:** Dinesh Rokaya

**Affiliations:** 1 Zarqa University, Az-Zarqa, Zarqa Governorate, Jordan

The authors have edited and improved the manuscript, and it can be accepted.

Are the rationale for, and objectives of, the Systematic Review clearly stated?

Yes

Is the statistical analysis and its interpretation appropriate?

Yes

If this is a Living Systematic Review, is the ‘living’ method appropriate and is the search schedule clearly defined and justified? (‘Living Systematic Review’ or a variation of this term should be included in the title.)

Partly

Are sufficient details of the methods and analysis provided to allow replication by others?

Partly

Are the conclusions drawn adequately supported by the results presented in the review?

Yes

Reviewer Expertise:

Prosthodontics, maxillofacial prosthetics, dental implants, dental materials

I confirm that I have read this submission and believe that I have an appropriate level of expertise to confirm that it is of an acceptable scientific standard.

# Response

**Published:** 2024-08-13

**Authors:** Vignesh Kamath

**Affiliations:** Department of prosthodontics, Manipal College of Dental Sciences Mangalore, Manipal Academy of Higher Education, Manipal, Karntaka,India,576104, Mangalore, Karnataka, India

Thank you for the acceptance

# Reviewer report: Reviewer response for version 1

**DOI:** 10.5256/f1000research.162458.r289857

**Published:** 2024-06-27

**Authors:** Dinesh Rokaya

**Affiliations:** 1 Zarqa University, Az-Zarqa, Zarqa Governorate, Jordan

This is an interesting article where the authors did a systematic review to study the antimicrobial properties of graphene on dental implants. However, the methodology is not clear which needs clarification and improvement, especially the search strategy. The abstract needs improvement. Finally, the manuscript needs revision and English language improvement. 

 Some comments on this research are as follows.

 Abstract

 The method section is not clear. It is better to add details.

 Introduction

 For the line “Surface coating of titanium dental implants is an essential aspect of implant dentistry, as it plays a crucial role in enhancing osseointegration, stability, and long-term success of the implant”. Add reference [Ref -1]

 Graphene coatings stimulate osteogenic differentiation, cell adhesion, and antibacterial activity. Add reference [Ref-2]. In these lines, there are no citations, so I recommend to add one.

 Add more to the statement with references “Graphene oxide coatings can improve the visibility of dental implants in imaging techniques like X-rays or CT scans, aiding in diagnosis and follow-up care.”

 Method

 The search strategy is confusing. Please add the total search search words used. In the Table 1, it is not good to mix the PICO and the search words. The authors can add PICO in a separate table.

Are the rationale for, and objectives of, the Systematic Review clearly stated?

Yes

Is the statistical analysis and its interpretation appropriate?

Yes

If this is a Living Systematic Review, is the ‘living’ method appropriate and is the search schedule clearly defined and justified? (‘Living Systematic Review’ or a variation of this term should be included in the title.)

Partly

Are sufficient details of the methods and analysis provided to allow replication by others?

Partly

Are the conclusions drawn adequately supported by the results presented in the review?

Yes

Reviewer Expertise:

Prosthodontics, maxillofacial prosthetics, dental implants, dental materials

I confirm that I have read this submission and believe that I have an appropriate level of expertise to confirm that it is of an acceptable scientific standard, however I have significant reservations, as outlined above.

## References

[1] : Contemporary Concepts in Osseointegration of Dental Implants: A Review. *Biomed Res Int* .2022;2022: 10.1155/2022/6170452 6170452 10.1155/2022/6170452 35747499 PMC9213185

[2] : Graphene for Antimicrobial and Coating Application. *Int J Mol Sci* .2022;23(1) : 10.3390/ijms23010499 10.3390/ijms23010499 35008923 PMC8745297

# Response

**Published:** 2024-08-03

**Authors:** Vignesh Kamath

**Affiliations:** Department of prosthodontics, Manipal College of Dental Sciences Mangalore, Manipal Academy of Higher Education, Manipal, Karntaka,India,576104, Mangalore, Karnataka, India

Thank you for all the valuable comments. We have responded to all the comments provided by the reviewer and made the necessary corrections. The updates are as follows:

1. Comment 1: Added the references suggested by the reviewer for the two sentences.
2. Comment 2: Modified the search strategy as per the reviewer's suggestions.
3. Updated and modified the reference list accordingly.

All changes have been made using track changes, and the new version of the manuscript is submitted for your review.

 Thank You
